# Benefits and barriers: a rapid-ethnographic study on the perspectives of potential and actual clients of Athens’ drug consumption room

**DOI:** 10.1186/s12954-025-01371-0

**Published:** 2026-01-26

**Authors:** Benjamin D. Scher, Nikolaos Poulopoulos, Christos Anastasiou, Benjamin W. Chrisinger, David K. Humphreys, Gillian W. Shorter

**Affiliations:** 1 Princeton University, School of Public and International Affairs, Princeton, USA; 2 University of Oxford, Department of Social Policy and Intervention, Oxford, UK; 3 National and Kapodistrian University of Athens, Department of Psychology, Athens, Greece; 4 European Network of People Who Use Drugs, European Union, Athens, Greece; 5 Greek Peer Network of Users of Psychoactive Substances, Athens, Greece; 6 Tufts University, Department of Community Health, Medford, USA; 7 Queen’s University Belfast, School of Psychology, Belfast, UK; 8 TreAdd Research group on Treatment and Addiction, Tampere University, Tampere, Finland

## Abstract

**Background:**

In April 2022, a new Drug Consumption Room (DCR) opened in Athens’ city centre. To date, no qualitative research has evaluated the operational strengths and weaknesses of the site from the viewpoint of people who use drugs locally, including those who use the DCR and those who use in street-based settings who do not access the DCR.

**Methods:**

Rapid-ethnographic fieldwork was conducted over a seven-week period. This comprised an initial five-week period of non-participant observation (≈ 200 h) followed by a community consultation regarding the research design and question protocols. Qualitative data were then collected through five focus groups with 24 regular DCR clients and 25 street-based interviews with non-DCR clients who consume drugs in street-based settings.

**Results:**

Regular DCR clients reported increased physical, structural, and emotional safety and increased connection with auxiliary health and social services and staff and peers. Those who did not use the facility could see potential benefits but noted several operational and contextual barriers including anticipated stigma within the service and contextual and operational barriers.

**Conclusion:**

Addressing DCR barriers could increase service access, reduce the presence and visibility of street-based drug use, and improve public health outcomes for people who use drugs in Athens. Indeed, some of these barriers have been addressed since the research was conducted (e.g. by expanding operating hours, increasing the number of staff with lived experience, offering on-site drug checking), illustrating the value of evaluating DCRs and subsequently adapting design and delivery based on the perspectives of actual and potential clients.

## Introduction

Athens experienced severe social and economic impacts from the 2010 global financial crisis [1] leading to a sharp increase in the number of people living in poverty, increased rates of urban homelessness, and increased HIV prevalence [2, 3]. Despite harm reduction interventions being scaled up during this period [4], Athens continues to face challenges. Scholars have evidenced how difficulties in delivering HIV prevention, including a lack of needle and syringe coverage during the COVID-19 pandemic and limited urban social housing availability contributed to a new surge in HIV cases [5, 6]. Additionally it has been documented how people experiencing homelessness in Athens’ city centre frequently face food and hygiene insecurity [7].

In April 2022, a policy window shaped by the urgency of safeguarding vulnerable populations during the COVID-19 pandemic [8], led to the expansion of several progressive drug and housing interventions, including: (1) the expansion of the city’s housing first programs, (2) the liberalization and expansion of national naloxone policy and (3) the opening of a drug consumption room (DCR). Nine years after the closure of Athens’ first DCR [9], and now with support from national and local level politicians [10], OKANA, who operate and oversee most of Greece’s drug services, opened a DCR. In addition to drug consumption supervision and overdose response, the site, colloquially called Steki 46, provides a range of on-site services as well as an off-site referral system for auxiliary health and social care programs. Like many DCRs globally [11], the primary aims of Steki 46 are to prevent and intervene in overdoses, provide harm reduction advice, and provide sterile equipment. By supporting people who use drugs within a medicalised setting the DCR works to reduce public drug consumption and drug-related litter in a community where public drug use remains visibly present. To engage with people, who at present do not access the service, the DCR is also the base for OKANA’s ‘street outreach team’ who advertise the service, offer equipment, sharps boxes, harm reduction and other health advice, and can make referrals to people in street-based settings across the city.

The building in which the DCR is housed additionally offers on-site primary healthcare, mental health services, social services, and retains the ability to refer off-site (see methods for more information on the DCR). As reported by Temenos et al. [12], primary healthcare and healthcare professionals more broadly play a limited role in detecting and addressing drug related harms within the Greek healthcare system. The most recent data available suggests that people who use drugs are largely self-referring to drug treatment and harm reduction services [13]. As implemented in other urban contexts, the integration of auxiliary healthcare into this site represents a direct effort to address this gap [14, 15].

The aim of this study was to provide immediate policy recommendations to OKANA on how to build on the strengths of the service and increase access to people who could benefit from the service locally. Most DCR evaluations rely on service user perspectives; here, we seek to expand understanding of the experiences and potential barriers faced by those who could benefit from the service yet do not access it [16]. This holistic understanding of Steki 46’s model of operation and service design, is developed with a range of perspectives including: (1) people who regularly attend the DCR, (2) people who use drugs locally who do not access the service, and (3) DCR staff. In this paper, we analyse the perspectives of DCR users and DCR non-users. The perspectives of staff will be reported elsewhere.

## Methods

### Study setting

This study took place at the Athens DCR and in the immediately surrounding neighbourhoods. This DCR is a medicalised model. It has an on-site general practitioner, nurses, psychologists, counsellors and social workers present to offer wrap-around support. On the ground floor there are twelve injecting booths and an inhalation room with space for up to four people. The ground floor has a medical room for the on-site medical staff, a kitchen, toilets, washing machines, showers and a courtyard garden for clients. On the second floor of the building there is a common room with board games, cards games, TVs, meeting rooms, a kitchen from which food is distributed to clients throughout the day, a cold-water station, a coffee machine, and offices for the on-site social workers, pro-bono legal workers, and employment assistance staff. The third floor has more offices for the various professional staff and the fourth floor is the central hub and main office of the ‘streetwork’ outreach team who use the building as a base for outreach trips across the city. Observations and fieldwork occurred throughout the building, with client focus groups and staff interviews taking place on the second floor. Interviews with people who use drugs who did not access the service occurred in street-based settings. These interviews were conducted in three separate open-air drug scenes, in locations where the ‘streetwork’ team conducted their outreach activities. These tended to be alleyways and urban parks with a high density of people consuming drugs, all within a 500m-2 km radius of the DCR.

### Rapid-ethnographic approach

This study used a rapid-ethnographic approach. Rapid-ethnography is defined by four distinctive characteristics [17] all of which we incorporated. One, the research must be carried out over a short, compressed or intensive period of time; we conducted intensive data collection over a six week period. Two, the research captures relevant social, cultural and behavioural qualitative data and is focused on human experiences, perspectives and practices; our question protocols were designed explicitly to understand people’s experiences, perspectives and practices in relation to the DCR. Three, the research engages with anthropological and other social science theories promoting reflexivity; reflexivity in relation to each of the researcher’s positionalities during fieldwork and data analysis were a primary focus and something we debriefed on as a research team regularly. Four, data must be collected from multiple sources (various stakeholders implicated in the topic of focus), using multiple modes of data collection and be triangulated during analysis; we conducted participant observation, focus groups with service attendees, and interviews with staff and people who used drugs in street-based settings, enabling triangulation of all reported experiences and perspectives. The strengths of this approach lie in its ability to produce research with a “nuanced understanding of lived experiences while prioritizing efforts to rapidly inform interventions and decisions that address urgent health and social issues” (Collins et al., 2020, p.384) [18]. In response to OKANA’s request that this research inform policy recommendations to improve service delivery and accessibility, rapid ethnography provided a flexible framework that could quickly capture the diverse views of people who use drugs locally through a variety of qualitative methods. For example, participant observation allowed us to organically document the interconnectedness of services offered in the facility, focus groups with regular service users documented the individual and collective experiences and perceptions of these services, and interviews with people who use drugs in street-based settings gave specific rationale for service non-attendance. Within a condensed timeframe, alternative methodological approaches such as client surveys or the use of service user data alone would not have captured the relational or experiential insights needed to ascertain potential areas of service improvement.

### Overview of methods

The methods of data collection were chosen specifically for their ability to rapidly observe and capture the ways in which clients and those who do not use the DCR, view and engage with the service. Data collection comprised seven weeks of fieldwork, including: (1) a community consultation, (2) five weeks (≈ 200 h) of participant observation and fieldnotes within the DCR, (3) focus groups with regular clients, (4) informal street-based rapid-ethnographic interviews with people who do not use the service and (5) semi-structured interviews with staff and management (which will be reported elsewhere).

### Research team and partnerships

This study was conducted as part of the doctoral work of BDS, with fieldwork led by NP, a graduate student in addiction studies at the University of Athens and CA, project manager with the European Network of People Who Use Drugs and the Greece based Peer Network of Users of Psychoactive Substances. Both researchers are local to Athens and have a deep knowledge of the local community and environment. CA has lived and living experience of OKANA service attendance and has worked as a peer researcher on several other related projects. GWS, BWC and DKH supervised the project, supporting design and analysis, meeting regularly with the research team online during fieldwork.

### Participant observation, recruitment and sampling

During the initial five weeks of participant observation NP immersed himself within the day-to-day operations of the DCR, on the ground floor and second floor (consumption and post-consumption spaces). During this period, he built rapport with clients and discussed the scope of the project and upcoming focus group dates with eligible participants. Since not all people who entered the DCR during this initial period had consented to the study, only generalizable fieldnotes were collected related to interactions, routines and contextual factors (e.g., what level of privacy did people have when sat at a booth, how long did people spend on average in each booth etc.). Whilst the primary aim of this initial phase was to build rapport, these observations also provided important context for the research team to interpret participant narratives (e.g. observations of withdrawal symptoms, mobility challenges, and daily service routines informed how the research team understood and probed emerging themes during focus groups and interviews). Observations were triangulated with what was reported in the more formal interviews and focus groups, and narratives were not accepted uncritically but understood in relation to thematic patterns described across data sources as well as what the research team directly observed during fieldwork.

Recruitment for focus groups was done through a combination of snowball and purposeful sampling [19]. People who signed up were encouraged to discuss the study with eligible members of their peer network. Eligibility was defined as: (1) aged 18 + years (2) used the DCR regularly, (3) provided informed consent and (4) could speak Greek. Participants in the street-based interviews were approached by CA who introduced the project to them. Those who wished to take part would move to a private location. NP would then conduct the interview. Interview eligibility matched focus groups other than participants had to also have experience of local street-based drug use.

### Community consultation, data collection and compensation

Following the five-week period of participant observation, CA led a consultation with six DCR clients. Here, the group discussed the research design and question protocols, and feedback was sought to develop the questions alongside the aims of the study, and to be trauma informed in line with best practice [20]. Rapid-ethnographic data collection was conducted over the final two-week period. Typically, in the morning NP conducted a focus group and, in the afternoon, BDS, NP, and CA would accompany the ‘streetwork’ outreach team to conduct street-based interviews before returning to conduct 1–3 staff interviews. These multiple methods of qualitative data collection afforded triangulation of findings [21].

Five focus groups were conducted which lasted 40–60 min and contained four to six people per group (total *n* = 24). Participants were aged 30–56 years (Mean = 44; SD = 6.4). One participant self-identified as Black, two preferred not to say, 22 self-identified as White. There were 7 females and 18 males, 9 were housed, 4 in temporary or emergency shelter accommodation and 11 self-identified as unhoused (Table 1). The question protocol was semi-structured, and participants were asked questions related to their experiences using the DCR, their views on operational policies and if they would add to or change anything about the services on offer within the facility. These included questions such as: “How often and why do you come to the DCR?”, “How would you describe your relationship with staff?”, “Which services within the facility do you tend to use?” and “Has the DCR impacted your life beyond the immediate harm reduction benefits of consuming drugs whilst supervised?”. Participants were recruited from the DCR by NP and CA, who would then accompany them upstairs to the private meeting room. Once the team had the desired number of people, CA explained the scope of the project, assisted people in completing the consent forms and demographic questionnaires. NP then conducted each focus group and ensured that the session was audio recorded for subsequent transcription and translation. Snacks, coffee, and soft drinks were provided during the sessions.

**Table 1:** Focus group participant demographics

Characteristic	*n*	%
*Age in years*		
Range	30–56	–
Mean (SD)	44 (6.4)	–
*Race/ethnicity*		
White	21	87.5%
Black	1	4.2%
Prefer not to say	2	8.3%
*Gender*		
Male	17	70.8%
Female	7	29.2%
*Housing status*		
Housed	9	37.5%
Temporary/emergency shelter	4	16.7%
Unhoused	11	45.8%

Ethnographic street-based interviews were conducted by NP, who took handwritten fieldnotes and wrote down verbatim quotes of importance. These quotes were then read back to participants at the end of interviews to make sure they accurately reflected the conversation. Interviews were semi-structured and followed a question protocol relating to their experiences of street-based drug use, their perceptions of the DCR and rationale for non-attendance. Questions included: “How would you describe your experiences of using drugs in locations like this?”, “Have you heard of the DCR, if so, what are your views on the facility?”, “Do you ever attend the facility, if not, why?”. These interviews lasted between 5 and 15 min. To support anonymity, no names were taken and the research team only gathered gender information. Participants in the community consultation/focus groups received €15 reimbursement; those participating in a street-based interview received €10.

### Ethics

Ethical approval for this study was granted by the University of Oxford on 11/03/2023 reference R84228/RE001 with internal ethical approval from OKANA. The approved, anonymised ethics protocol is hosted on the Open Science Framework (DOI OSF: 10.17605/OSF.IO/VS4AT). OKANA also provided logistical support through: (1) office space for focus groups, (2) snacks and drinks for clients during the sessions, and (3) safeguarding support for street-based interviews but were not involved in research conduct, analysis, or conclusions. During focus groups and street-based interviews, staff were not present and could not hear what was being said.

### Consent to participate

All participants gave informed written consent after reading and having the opportunity to discuss the information sheet with CA. During these discussions participants were made aware of the ways in which discussions were recorded, how the generated data would be used and that their participation was voluntary and that they could stop at any time and for any reason. Ethical considerations specific to this study context and participant group were frequently discussed by the research team based on their experiences with similar research populations. Such discussions included questions how to make participants feel comfortable discussing the delivery of the very service in which the focus group is taking place. For this we decided that as a peer, CA, would spend time ensuring that all participants understood how the data would be anonymized; that care would be taken to ensure that identifiable quotes would be removed from any of the reported data.

### Translation and data analysis

Following data collection, all transcripts, which were in Greek, were translated into English. Microsoft Teams translation software was used to generate initial translations, which were subsequently checked and amended by NP, a bilingual researcher with professional experience working in both English and Greek. English transcripts were then uploaded to NVivo QSR 2023 software enabling analysis. Data analysis comprised of the Braun & Clarke [22] six stage reflexive thematic analysis, commencing with a process of familiarization with the data, whereby NP and BDS read transcripts and fieldnotes, wrote analytic memos and collectively developed a thematic codebook to work systematically through the data. The coding process involved identifying relevant and meaningful excerpts related to the research questions within the transcripts and fieldnotes, seeking out passages which spoke to novel concepts, grouping related codes into broader analytical categories and discussing variations or contradictions between the various codes developed by one another. These codes were then reviewed by GWS, DKH, BWC and CA who refined and challenged the initial code definitions. BDS and NP subsequently went back and applied the final codebook until all the data were organized into a final coding framework that was approved by all authors [23].

### Results

Following the coding of transcripts, three central themes were generated, each with several sub-themes related to the perceived and experienced benefits and barriers of Athens’ DCR. Themes incorporate the views of both daily DCR service users and people who use drugs in street-based settings (see Table 2).

**Table 2:** Themes and sub-themes of the views of people who use drugs (who do and do not use the Athens DCR)

Theme	Sub-theme
1. Safety	1.1. Physical safety
	1.2. Structural safety from police and criminalization
	1.3. Safety from stigma and desire for privacy
2. Connection	2.1. Access to basic necessities
	2.2. Built relationships in the DCR
3. Barriers	3.1. Anticipated stigma
	3.2. Operational barriers
	3.3. Physical barriers

## Safety 

### Physical safety

Participants regularly contrasted the environment of the DCR to that of the ‘piazzas’, the local slang used to describe the urban open-air drug scenes. This comparison often centred around the physical safety afforded by the DCR, as highlighted by one participant who described the relief experienced when accessing the service:

*“The benefits, well it is a breath of fresh air away from the road, from the piazza, it’s a safe place.”* (Regular DCR Client, White, Male, 40-45 yrs, Housed)

Expanding on this, another participant described how the DCR offers respite for people who use drugs and experience homelessness:

*“It is a place that provides security. Many younger people without shelter cannot sleep at night. They run all night right and left and come here during the day, they use early [in the day] and many times after using you see them get sleepy. They're tired, they're finished, and you see them come over here and look to get a little sleep...in a sheltered space they know won't take away their things.” *(Regular DCR Client 2, White, Male, 40-45 yrs, Unhoused)

The ability to rest without fear of being robbed was seen as a significant benefit to many. This was reiterated by non-DCR users: *“Over there, there is security, while out on the street there is none and you can be easily robbed.”* (Non-DCR User, Interviewee 8, Male). Alongside providing a safe environment, the knowledge there would be a swift medical response to keep people alive during an overdose was commonly cited as a primary benefit and reason people attended the DCR: *“You know that there are doctors here so there is no chance of you dying.” *(Regular DCR Client, Black, Male, 45-50 yrs, Unhoused).

The non-verbal reassurance provided by the presence of staff, even when no intervention was needed, was a central aspect of participants’ sense of security and trust in staff: *“When something happens to me they [staff] are there to help me. [Even] just watching me, I feel safe.” (Regular DCR Client, White, Male, 35-40 yrs, Unhoused)*. Safety was also discussed beyond that of physical safety, with this participant associating safety with the harm reduction equipment available within the DCR: *“With the scanner I can see the veins that are to be punctured. This is very helpful.” (Non-DCR User, Interviewee 21, Male)*. Despite street-based interview participants not regularly accessing the DCR, there was a general awareness and understanding of the ways in which the DCR would offer increased safety from overdose, theft, and injection-related harms.

### Structural safety from police and criminalization

For many, protection from police encounters and the risk of criminalization was a key motivator for attending the DCR:

*“The fact that there is a place...where we will not be bothered by the Law is a good thing...it is a place where I can use without the constant fear of the police.”* (Regular DCR Client, White, Female, 35-40 yrs, Housed)

Supporting this statement, this participant described how the fear and anxiety of police harassment and rushing the injection process to avoid police detection lead to physical injuries:

*“The benefit is that you're not being chased by the police, the stress of use especially if it's intravenous and you see the cops in front, you can easily do something wrong, as I suffered. I put a pinch of sisa [methamphetamine] together and then saw the cops in front of me [and rushed]...I have had an abscess from that which is still slowly recovering.”* (Regular DCR Client, White, Male, 35-40 yrs, Unhoused)

Such injuries are rare when an individual can take their time in the DCR. Mistrust of the police also related to whether they would respond appropriately in the event of an overdose:

*“This is where the DCR is needed because when something happens inside, staff will...help you…if I’m outside, I don't know if the police will call the ambulance.”* (Regular DCR Client, White, Male, 35-40 yrs, Temporary Shelter Accommodation)

Police were perceived as not prioritizing the health and safety of people who use drugs. This fear extended to various other aspects of criminalization such as arrest or processing through the courts, which would also have implications such as the confiscation of drugs, and potential withdrawal:

*“The security you get here is that you will not be taken to court, your fix will not be taken. You don't know how much I...do to get my dose, and then it just gets taken away.”* (Regular DCR Client, White, Male, 50-55 yrs, Unhoused)

In contrast, many expressed a sense of relief knowing they could use the DCR without fear of arrest or harassment, specifically contrasting interactions with police (and other community members) to those they experienced with staff: *“There you feel safe from the residents and from the police. There I can ask about medical problems and they treat us like human beings.” (Non-DCR User, Interviewee 15, Female)*.

### Safety from stigma and desire for privacy

There was agreement from regular clients that by ensuring privacy, the DCR provided emotional safety from stigma; there is respect and dignity not often experienced in street-based settings and everyday encounters with the public. Contrasting the privacy offered within the service, this participant described the emotional discomfort experienced when witnessed using drugs in street-based settings:

*“For me I come here [because] I don't like to use on the street. I don't want everyone who passes by to see me.*” (Regular DCR Client, Black, Male, 45-50 yrs, Unhoused)

Participants consistently described the relief of not using in street-based settings and particularly how the DCR helped reduce the visibility of drug use on the street, particularly around children:

*“Children now don't have to see me. I…come and do it [use drugs] here because otherwise I do it on the sidewalk. Imagine being with your child...and explaining to them what that is, having that bad conversation. I come here in the morning hours more. Why? Because I'm on the street and homeless I can't sit out on the step, because the police are pushing me away, the shops are kicking me of their steps...especially now with tourists. In the morning, when the whole world is out...I'm ashamed, so I see this place as a shelter to hang out, to take some time to myself and drink some coffee”*. (Regular DCR Client, White, Male, 40-45 yrs, Unhoused)

Such reflections where participants highlight both the relief of no longer using in street-based settings and exposed to uncomfortable encounters with the public, accompanied with awareness and assertion of broader perceived benefits to the community (eg., reducing the visibility of public drug use, safeguarding children), demonstrates the broad understanding people who use drugs hold with regards to the potential benefits of the DCR.

## Connection

### Access to basic necessities

When asked what they valued at Steki 46, regular attendees highlighted the tangible benefits to their daily lives through being able to access auxiliary medical services, food, hygiene facilities and other basic necessities:

*“It is the clean space, medical care and toilets, because I am homeless [and] the main problem is [accessing] toilets. The bathroom, the washing machine…the supervision of the doctor is a bonus on top of that. It all helps immensely. Also, the referrals to the hospital, to legal aid…they all help.”* (Regular DCR Client, White, Male, N/A yrs, Temporary Shelter Accommodation)

Many who participated in focus groups were either in situations of homelessness and/or severe financial precarity. In this context, the provision of regular snacks, sandwiches, coffee, and other donated meals was an essential resource:

“*This is a very big help. Coffee for example…finances are difficult, [this] is what will make the most difference to the people outside. Some days when I am broke, it really helps me to come and eat a cheese pie…lunch food not just snacks.”* (Regular DCR Client, White, Male, 40-45 yrs, Housed)

For individuals without a stable income, items like coffee or a sandwich made a meaningful impact. For individuals with pre-existing health conditions such as diabetes, access to food at the DCR was both a convenience and a necessity. One participant reflected on how the staff ensured he received this support:

*“[I am diabetic], when my blood sugar drops they give me something sweet. They make sure I…stay safe. I really like that. The people here help immensely…I am pleased to be a member here.” *(Regular DCR Client, White, Male, N/A yrs, Temporary Shelter Accommodation)

These essential provisions contributed to regular clients’ perception of care within the DCR, highlighted by this participants description of themselves as a ‘member’, a term encompassing a sense of belonging to the service. Whilst many began by describing the immediate, practical benefits of these auxiliary services, their accounts frequently expanded to encompass other more formal, institutionalized forms of care they were able to access through support by staff:

*“For so many years I couldn't cut down, but the guys that work here helped me. They also encouraged me into a treatment program....this place has made me realise that I can really make an effort to escape this life.”* (Regular DCR Client, White, Male, 40-45 yrs, Unhoused)

DCR staff supported participants’ personal efforts toward change, particularly in relation to drug treatment. Previously dismissed as unattainable or unappealing by many clients, the DCR was a place where the idea of recovery, reducing consumption or making healthier choices in relation to one’s drug use, became a tangible goal.

### Building relationships in the DCR

Many participants described the positive social connections created in the facility. Field notes captured how only half an hour from when the DCR would open in the morning, the second-floor lounge was nearly always busy with clients watching TV, playing cards, and Tavli (Greek backgammon) with staff and clients having coffee and cigarettes on the balconies. Engaging in activities with peers and staff had positive effects, including alleviating boredom, described as a common trigger for substance use:

*“Here you will find other people to socialize with...do other things that fill up your time because many times you drink or use drugs out of boredom.” *(Regular DCR Client, White, Male, 50-55 yrs, Housed)

Many regular attendees also described positive relationships with staff in the context of them connecting clients to services as well as the care and respect they exemplified when working with clients:

*“In the beginning, I wondered about why it exists, but there happened to be a girl, a member of staff, who showed great interest [in me] …She helped me to deal with anything I needed, from benefits to whether I was interested in going into a detox program.”* (Regular DCR Client, White, Male, 30-35 yrs, Unhoused)

The ability of staff members to guide clients through complex service systems, whether related to benefits, healthcare, detox or drug treatment programs, was a frequent theme in participants' accounts and commonly described:

*“I got my ID through the DCR with the social worker, she was very helpful. She is much more helpful than if I would have gone alone to the office…that’s another good thing…when there is someone in front of you who...sees that you know someone [a member of staff], everybody behaves a lot differently. Because they are very...discriminatory towards us in hospitals…we are not taken seriously.”* (Regular DCR Client, White, Male, 50-55 yrs, Housed)

This description of staff advocacy speaks to the critical role played by staff in addressing the broader social inequalities that impact clients’ ability to access health and social services. Participants described how staff with lived experience were particularly effective in communicating with clients:

*“The staff are very flexible, there are also ex-users and this plays a big role. They understand us even better and are very flexible with us and polite.” *(Regular DCR Client, White, Male, 40-45 yrs, Housed)

Supportive staff played an important role in clients' ability to access services, feel respected in potentially hostile environments, and establish a sense of trust with the facility. For many, these positive relationships were central to their continued engagement.

## Barriers

### Stigma

For some, the formality of the DCR, combined with concerns about being judged within the service, acted as a deterrent, as noted by this participant: *“They feel more at home in the piazza. They feel like they are being mocked or feel a bit uncomfortable [in the DCR].” (Regular DCR Client, White, Male, 35-40 yrs, Unhoused)*. For individuals who feel more at ease in the less regulated spaces of the piazzas, the transition to the DCR, which can appear more clinical or formal, may exacerbate feelings of alienation: *“I consider the space like a dentist’s office...that doesn't work for me.”* (Non-DCR user, Interviewee 9, Male). This participant for instance who now attends the service regularly, recalled how *“What was difficult...was actually showing the drugs at first*”. (Regular DCR Client, White, Male, 35-40 yrs, Unhoused). The act of presenting drugs for consumption in a supervised setting can evoke feelings of vulnerability and anxiety, as people using the facility may fear judgment from staff or peers; and it diverges from street-based practices where drugs can remain hidden. This sentiment was corroborated by a participant who highlighted the sense of apprehension about entering an unfamiliar space without peer support: *“I don't know anyone there and I don't feel comfortable going alone.” (Non-DCR User, Interviewee 3, Male)*.

### Operational barriers 

The overarching system of surveillance in the DCR, though for the purpose of reducing and responding to risk (e.g., overdose response), was acknowledged by many through comments such as: *“in the DCR there is surveillance” (Non-DCR User, Interviewee 3, Male)*. A concern and cause of discomfort voiced by several participants was the feeling of being watched, either by staff or through cameras when using drugs: *“There is the fear that there are cameras watching me there, I would like there to not be any.” (Non-DCR User, Interviewee 23, Male)*. This system of surveillance and risk reduction extended to specific moments within the intake and consumption process. For example, people who did not use the service cited the length of time from arrival at the facility to when you can consume drugs as a significant barrier. These experiences were again framed within the context of the unpleasant experiences of withdrawal:

*“I have been once and only once. It is a time-consuming process. When I'm...going through withdrawals or very high I don't feel like spending it [time] there.”* (Non-DCR User, Interviewee 18, Male)

The need for immediate relief, and the time needed for the structured intake process to occur was described as a clear barrier to service engagement. Attendees valued the medical care available, however, for some, the time spent checking in with a doctor each time they wished to use the site was a barrier:

*“When you are sick, you don’t have the time to do paperwork, to see a doctor or any of those things, you don’t have time for that.”* (Non-DCR User, Interviewee 6, Female)

While medical oversight is a core feature of the DCR, these experiences highlight how it may not serve the immediate needs of their client group. There were also acknowledged differences in the experience of preparing and consuming drugs in the DCR compared to street-based settings:

*“Not everyone can fit into one mold, workers can't understand users. I…enter and they ask me what substance I have on me. I often use with my friends by doing small transactions but inside they don’t let us do any transactions or share. It is very different to how we would use on the street.”* (Non-DCR User, Interviewee 12, Male)

*“In the DCR there are many limits, there is surveillance, documents that you have to fill out. While on the street there are no limits, no rules” *(Non-DCR User, Interviewee 3, Male)

These participants explain how the DCR environment did not mirror socially driven consumption practices particularly in relation to sharing or exchanging drugs, a sentiment echoed by others who found that restrictions around mutual aid, such as being able to inject or assist a friend during consumption, was a reason for service avoidance:

*“I've went when it first opened. Often the ones who don’t want to go, it's because here in the square or on the street there is more help. There [the DCR]...your friend cannot inject you, while here...people can do things like that.”* (Non-DCR User, Interviewee 19, Female)

Additionally, as is common in other international DCR contexts, certain higher-risk injection practices were not permitted. This contributed to people using drugs street-based settings where such injecting practices were not controlled or managed:

*“Over there, it is forbidden to shoot in the neck or the artery. One may not want to go there because it is forbidden.”* (Non-DCR User, Interviewee 10, Female)

One final and notable operational barrier which was discussed was the service restriction for people who were in opioid substitution treatment programs (OST). This was observed during the ethnographic fieldwork and noted by this participant: *“Many users do not come because...if you are in an OST program you cannot come to use.” (Regular DCR Client, White, Male, 50-55 yrs, Unhoused)*. Someone who uses drugs in street-based settings explained they are now disqualified from accessing the DCR despite having built important relationships with members of staff:

*“The staff there are like my family looking after me. But since I’m in a substitution program I can't go there...it's forbidden.”* (Non-DCR User, Interviewee 20, Male)

The sense of disconnection experienced by this participant highlights how important the social aspects of the DCR can be for individuals who may otherwise be socially marginalised.

### Physical barriers 

Despite the many benefits which framed beliefs around why people attend or should attend the DCR, several physical barriers were described. Distance was perceived as an important factor which may dissuade people from attending:

*“I, [live] by Victoria square which is ten minutes away, but for someone who lives far away I don’t think they will come to the DCR. So it would be good if more existed.”* (Regular DCR Client, White, Female, 45-50 yrs, Unhoused)

For individuals interviewed in street-based settings, the urgency and necessity to alleviate withdrawal symptoms was often cited as the primary reason for not being able or willing to commute long distances once in possession of substances:

*“If I am sick [from withdrawal], I use in the first place that I can find…if I am sick I will use anywhere...500m seems like 500km when you are sick”* (Non-DCR User, Interviewee 6, Female)

Whilst issues related to mobility were commonly observed by the research team during fieldwork, regular attendees additionally identified a lack of awareness amongst the local population of people who use drugs as a barrier to the service. Participants explained that beyond simply knowing about the service, people needed to be given a better understanding of exactly what takes place at the DCR:

*“A lot don't even know about it, they might have heard it as an idea, but they haven't come to see for themselves. It would be great if one day staff came to pick them up to take them…or [came] to show them pictures of the DCR at the piazza. They should invite them...they are suspicious and don’t dare to take the step to come over here and see.”* (Regular DCR Client, White, Female, 40-45 yrs, Housed)

*“When you are homeless, you generally have a lot of phobias, that's why people can be suspicious. Especially to give one’s name, for it to be written down, even during intake people may wonder, why do they want to see the drugs I have?”* (White, Male, 45-50 yrs, Temporary Shelter Accommodation)

These reflections underscore the notion that simply having a service available is not enough; active outreach is necessary to break down the barriers of suspicion and unfamiliarity. When the research team interviewed people in street-based settings, these sentiments were echoed:

*“Many people don't know about it. Some may think that the police are cooperating and there may be a check if you go to this place.”* (Non-DCR User, Interviewee 16, Male)

The recurring theme of safety, both physical and emotional, emerges here, once again emphasizing the need for the DCR to communicate that the service will treat clients with dignity, respect and ensure anonymity.

## Discussion

The overarching narrative from this study was that the Athens DCR helped to achieve the stated objectives of responding to overdose and connecting more people to on-site and referred auxiliary, health, social and drug treatment services. Despite this, there remains a visible community of people who use drugs in street-based settings who do not engage with the site [24]. People who regularly attended the DCR, many of whom experienced housing and financial insecurity and substance dependence, experienced positive outcomes echoed in the international literature [11, 25, 26]. Regular clients also spoke to potential barriers that may prevent others from accessing the service – opinions supported by the experiences and perceptions of people who used drugs in street-based settings. Non-DCR users also spoke to additional barriers that regular service attendees had not mentioned such as the DCRs distance from street-based from drug scenes, fear of surveillance and lack of anonymity within the DCR, suspicion around on-site rules and service restrictions when concurrently enrolled in an OAT program. Despite this, non-DCR users had an awareness of the positive outcomes associated with DCR attendance. If the identified physical and operational barriers related to specific policies and design features of the Athens DCR can be adapted to meet the needs of the population currently not accessing the site, there is an evidenced desire from local non-DCR users to access the service to receive those benefits.

The results from this study align with the positive outcomes described in existing qualitative evaluations of fixed-site DCRs. Firstly, the primary aim of the DCR is to supervise drug use and manage overdose risks before, during and after the consumption event – in essence, to keep people safe [11]. This perceived feeling of safety, particularly around ‘staying alive’ [27, 28] was discussed at length by participants and demonstrates an interest and motivation of clients to manage their health [11, 25], 29– [31]. Clients’ conception of ‘safety’ goes far beyond the traditional public health metrics used to define safety. Instead clients operationalise the term in relation to environments and situations in which there is an absence of structural violence, protection from police and criminalization, and as refuge from the stigma experienced within street-based consumption environments. Privacy eliminated the need to rush the injection process, hide out in unsanitary and secluded drug consumption environments, and safeguarded against the threat of physical violence and stigma; all reoccurring themes across a range of qualitative studies of urban drug scenes [10, 32–41].

Food insecurity and a lack of access to basic hygiene facilities is a significant issue for people experiencing homelessness in Athens [2, 42, 43]. Our participants described how the DCR provided food, showers, washing machines, and toilets; and how elsewhere these needs are not met. The value placed on these services speaks to the level of need for people’s most immediate basic necessities of safety, food, hygiene, housing – all unrelated to their drug use. Thinking about the concept of intersectionality and in particular the intersectional risk environment [44], many of the clients of this facility experience intersecting forms of vulnerability (e.g., food insecurity, homelessness, mental and physical health issues, etc.), With this in mind, Athens’ DCR is an example of how enhanced or expanded harm reduction models can integrate responses to poverty and homelessness, in turn functioning as inclusion health interventions [45]. For individuals who move from the DCR into treatment (be that abstinence-based or opioid substitution therapies) and who lose access to the DCR, it is unclear where they would access this auxiliary support.

The second floor of the building, where clients can move onto once they have finished in the consumption space supports and facilitates increased feelings of socialization and belonging. Here, individuals relax with peers without the risks or fears that arise within street-based environments. The design choice to include a large space for relaxation post consumption, with no time limit regarding duration of stay differs from international contexts, however, was repeatedly emphasized as a key part of the service. Such environments reduce the likelihood of congregation on the street and support broader behaviour change and wellbeing [27, 46, 47]. Finally, participants spoke to reductions in street-based drug use which they saw and attributed to the presence and availability of the DCR.

Given the multiple benefits of Athens’ DCR, it is equally important to highlight the perceived barriers of people who could benefit from the service, yet who do not. In our interviews with non-DCR users, many of the stated barriers were contextual, however, others were very much a product of policies connected to the medical model applied at the DCR. As noted by Urbanik and Greene [16], barriers to DCR use are contextually specific, and therefore site-specific research is required for individual services to adapt design and increase access. Here, we group them under two categories of barriers: (1) operational barriers, and (2) physical barriers. For each barrier, we make recommendations which could lead to greater use of the DCR.

A primary barrier was the distance of the service from Athens’ ‘piazzas’; beyond consuming drugs with peers in these locations, people are often unable or unwilling to commute long distances to the DCR. Mobile DCR units which go out to these existing drug scenes could be effective [11, 48]. DCR attendees suggested there may be a lack of awareness regarding the operational policies of the DCR amongst the wider population of people who use drugs, and this led to apprehension about attending. More direct outreach campaigns, preferably led by peers, would help expanded local knowledge around the DCR, make people feel more at ease and increase access. This phenomenon is common, an understanding of the specific practices of harm reduction settings, can be a barrier to attendance elsewhere [11, 49, 50]. Several people, both who attended the DCR and did not, perceived self and actual stigma as a barrier. Some were unsure how they would be treated by staff or how they would feel being around professional, non-peer staff members. Recent research examining the perceptions of staff from drug services in Athens, has suggested some stigmatising attitudes towards people who use drugs [12]. Whilst this may not be the case for staff in Athens’ DCR, including peer workers as active members of the staff team (e.g., to welcome new clients) is an evidence-based approach which would make both the intake and general service of a DCR a less intimidating experience [51–53].

Interviewees cited the fear or being recorded or watched over during the consumption process as a barrier. This finding has been noted elsewhere and has led to DCRs being theorized as sites of governmentality [54–56]. Scholars have described how the biomedical guise of harm reduction leads to behaviour being monitored and individuals encouraged towards specific consumption practices. Indeed, the DCR’s emphasis on safety may deter individuals who equate privacy with freedom. Whilst supervision and surveillance is the central component to keeping people safe in supervised consumption facilities, making small adaptations to DCR policies, such as having a peer worker do the frontline supervision, with clinical staff in the background to intervene should an overdose occur can make individuals feel more relaxed and improve the general atmosphere of sites [52, 57, 58]. Additionally, communicating that people’s personal information and details of service attendance (i.e. frequency of attendance, substance consumed, etc.) will not be passed on to other health and social care agencies, and remains anonymous may also have a positive impact on service uptake and client outcomes as it has been in other services [59].

As noted by Urbanik & Greene [16], the emphasis on risk reduction within DCRs may be at odds with the needs of people who could benefit from the service. Issues arise when the environment and permitted practices within DCRs do not reflect the positive elements of socialization, pleasure or consumption ritual performed outside facilities [60–63]. Non-DCR users specifically cited the extended time to arrive at the site, fill in paperwork, meet with the doctor and then access the consumption space as a deterrent. Where possible, policies could be reviewed to acknowledge substance withdrawal symptoms whilst maintaining operational licences. Where efficiencies are possible, this will avoid unwanted situations in the DCR where individuals leave and consume drugs in street-based settings or isolated locations with risks to the individual and the public. Several other restrictions around the consumption process existed, for example: no sharing of drugs, no assisted injecting, no sharing of booths. Whilst these restrictions are typical of medicalized DCRs [16, 51, 64, 65], participants in Athens cited such policies as factors dissuading access. For example, during the intake process which took place upon each use of the service, the doctor would ask what and how much an individual was about to consume. A ‘harm reduction conversation’ would ensue. Here the doctor would sometimes recommend the person take less than planned or split their dose into smaller batches. Such conversations about the nature and quantity of substances pre-consumption have been reported elsewhere [66]. Although this was not mentioned during interviews, it was described during staff interviews. The power dynamics present in conversations between medical practitioner and potential DCR client are unlike those which could occur on the street and could dissuade people already apprehensive around the DCR experience.

Finally, several individuals reported that as they were enrolled in OST programs - a key evidence-based harm reduction tool - they were prohibited from accessing the facility. Whilst the purpose of OAT is to support individuals in achieving their treatment goals [67, 68] and reduce overdose risk by halting their consumption of drugs from the illicit market, this restriction also meant that an important support system of socialization and auxiliary services was cut off from this client group. This service barrier has not been widely reported on in other international contexts. Additionally, the subsequent inability of these clients to access the DCR when enrolled may lead some individuals to use in riskier, unsupervised settings, undermining harm reduction objectives of the service. Amending this policy could be considered to expand access to the DCR and auxiliary health and welfare services.

### Policy implications and study significance

A significant proportion of non-DCR users understood the ways in which the DCR could benefit them by reducing overdose risk, safeguarding from physical and structural violence common on the street, increasing access to auxiliary services and offering privacy during the consumption process. With this awareness in mind, we believe that implementing the recommended changes to DCR policies outlined here could effectively reduce the current barriers. This could allow more people to benefit from the DCR and decrease the prevalence of street-based drug use currently present within Athens’ city centre [24]. This work illustrates how a medicalized model can unintentionally generate new forms of exclusion under the guise of enhanced care. These findings underline the importance of not treating DCRs as a uniform intervention, but rather as highly context-dependent services whose operation is shaped by local health systems, policy frameworks, and deeply engrained sociocultural attitudes towards drugs and people who use them. Attending to these differences is vital if DCRs are to remain accessible to those who could benefit most.

### Limitations

We explore participant perspectives at a specific point in time (May/June 2023). It is important to note that views may change, especially given the service has adapted certain policies, such as expanding their operating hours, increasing the number of DCR staff with lived experience, increased numbers of mobile DCRs in Athens and on-site drug checking services, all which may have increased access to the site and altered client perspectives. As previously mentioned, transcripts were translated from Greek to English. Although this was to a professional standard, there may be some nuance and context which was lost in how people communicated. Our findings may also be subject to selection bias, with participants potentially holding different perspectives to individuals who declined to be interviewed or could not be reached [48]. This limitation is important to consider, as it may affect the diversity of viewpoints captured in our study. We did not explore the identities of our participants in detail here (e.g., how being a particular gender, race, or other characteristics might have influenced the findings). The role of intersectionality is important, underrepresented groups should be oversampled (e.g., here we had limited black individuals, and should be explored in future research in Greece and beyond). Finally, the views of people who access services are context specific and highly localized, our findings may not represent the realities of other communities in which DCRs are implemented and future research should look to assess benefits and barriers in other contexts as a way of adapting local DCR policies.

## Conclusion

This study highlights both the current successes and limitations of Athens’ DCR. Whilst the facility is undoubtedly preventing and responding to overdoses, providing essential health and social care services and reducing the impacts of structural violence for those who experience homelessness or who would otherwise consume substances in street-based settings, there remains a substantial number of people who do not access the site who use drugs in street-based settings. These participants identified physical and operational barriers, such as restrictive consumption practices and the timeliness of the intake and consumption process as factors which discouraged them from accessing the DCR. Addressing these barriers through more flexible policies, expanded outreach and communication of the DCR and integrating peer workers could increase service access. More broadly, adapting these policies to be more reactive to the needs of local people who use drugs could reduce the presence and visibility of street-based drug use and improve public health outcomes for people who use drugs in Athens

## Data Availability

The approved and anonymised ethics protocol is hosted on the Open Science Framework (https://osf.io/vs4at). Data is available from authors upon reasonable request.
